# Investigation of the Optical Properties of a Novel Class of Quinoline Derivatives and Their Random Laser Properties Using ZnO Nanoparticles

**DOI:** 10.3390/molecules27010145

**Published:** 2021-12-27

**Authors:** Abdulrahman I. Almansour, Natarajan Arumugam, Saradh Prasad, Raju Suresh Kumar, Mohamad S. Alsalhi, Manal Fahad Alkaltham, Haya bint Abdulaziz Al-Tamimi

**Affiliations:** 1 Department of Chemistry, College of Science, King Saud University, P.O. Box 2455, Riyadh 11451, Saudi Arabia; almansor@ksu.edu.sa (A.I.A.); sraju@ksu.edu.sa (R.S.K.); 441203407@student.ksu.edu.sa (M.F.A.); 2 Department of Physics and Astronomy, College of Science, King Saud University, P.O. Box 2455, Riyadh 11451, Saudi Arabia; srajendra@ksu.edu.sa (S.P.); malsalhi@ksu.edu.sa (M.S.A.); haaltamimi@ksu.edu.sa (H.b.A.A.-T.); 3 Research Chair for Laser Diagnosis of Cancers, Department of Physics and Astronomy, College of Science, King Saud University, P.O. Box 2455, Riyadh 11451, Saudi Arabia

**Keywords:** imino quinolines, photochemical properties, computational studies, random laser, ZnO nanoparticles

## Abstract

Quinoline Schiff bases display potential applications in optoelectronics and laser fields because of their unique optical properties that arise from extensive delocalization of the electron cloud, and a high order of non-linearity. In this context, a new class of conjugated quinoline-derivative *viz*. *N*-(quinolin-3-ylmethylene)anilines were synthesized from 2-hydroxyquinoline-3-carbaldehyde in two good yielding steps. The ability of these imines to accept an electron from a donor is denoted by their electron acceptor number and sites, which is calculated using density functional theory (DFT). The optical properties such as FT-IR, Raman, UV-VIS, and EDS spectra were calculated using TD-DFT, which also provided the energy gap, HOMO-LUMO structure. The optical properties of the synthesized imino quinolines were experimentally studied using photoluminescence and absorption spectroscopy. The properties such as Stokes shift and quantum yield were calculated using experimental data. Furthermore, the compound bearing a methyl group on the aryl ring and ZnO nanoparticles (hydrothermally synthesized) were dissolved in toluene, and optically excited with a 355 nm nanosecond laser, which produced a random laser.

## 1. Introduction

The discovery of a new class of highly rigid and conjugated organic molecules is fascinating in basic, applied science, communication, pharmaceuticals, construction, medical and military application [1,2,3,4,5,6]. In addition, organic molecules have found application in biosensors [1], sensors [2], light-harvesting [3], memory devices [4], bioimaging [5], and catalysis [7]. Most importantly, they have found broad application in optoelectronics, such as organic photovoltaics (OPVs) [8], organic field-effect transistors (FETs) [9], organic light-emitting diodes (OLEDs) [10], organic thin-film transistors (OTFT) [11], non-linear optical devices [12] and lasers [13]. As a surface light source, the OLEDs are widely used to build flexible displays, televisions, storage devices, solar systems, and other portable devices. These materials can be easily manufactured inexpensively, with higher energy efficiency than other inorganic materials. In particular, organic materials have contributed significantly to the development of dyes as laser materials, and have proven to be an excellent media for laser action from 350–900 nm with continuous tunability. The dyes have also been pumped using a continuous wave (CW) mode by Ar ion/Kr ion gas lasers. Two other significant advantages of these dyes are that they can work in ASE (amplified spontaneous emission) mode [14], and generate picosecond and femtosecond pulses with a suitable mode-locking design. Such dye lasers are indispensable tools in the basic and applied sciences, including cancer treatment (photodynamic therapy).

Although studies have been carried out on organic dye lasers in gas, liquid and solid-state phases, these lasers always perform best in the liquid state. In their liquid state, the heat associated with the laser process can be easily removed by a rapid flow of the dye solution. Due to the constraint for CW laser action, the dye must flow as a thin jet [15]; however, there are practical disadvantages of liquid dye laser systems, which are associated with the toxicity and flammability of several dyes and the need for large volumes of organic solvents. Furthermore, dye lasers are not easily transportable for field studies, which has resulted in an increasing interest toward the development and implementation of solid-state dye laser (SSDL) systems.

In the past few decades, rhodamine 6G and pyrromethene complexes have been used as solid-state dye laser materials because of their planar geometry with extended conjugation, and highly fluorescent nature. Since these materials have heteroatoms in rings such as oxygen and nitrogen, and are soluble in polar organic and water-soluble solvents, these dyes are more photochemically stable to withstand a few hundred thousand laser shots, and have more lasing ability. Nevertheless, the availability of dye-based lasers is very limited. Random lasers are an unusual type of laser called mirrorless lasers, which do not produce amplified spontaneous emission (ASE). The laser is achieved due to mesoscopic optical gain from synergic scattering on the surface of a nanostructure. Due to the simplicity in building this laser, it has many potential applications [16].

In this connection, quinoline is an attractive planar aromatic ring system that has well-known optoelectronic applications and high electroluminescence efficiency [17]. In addition, these derivatives possess diverse pharmacological activities, including antibiotic, anticancer, antioxidant, antimicrobial, anti-TB23, cardiovascular, receptor antagonists, anti-inflammatory, antiplatelet, NK3 receptor antagonists-II, selective estrogen receptor modulators (SERMs) and protein kinase inhibitor qualities. Quinoline Schiff bases particularly exhibit these broad pharmacological properties [18,19,20], and act as a fantastic optical storage device due to their extensive delocalization of the electron cloud and high non-linearity. The ability of a compound to accept electrons from a donor is characterized by its electron affinity (EA); this has been investigated by DFT studies, where the nature of the molecules was understood [21,22,23,24,25].

In this context, we have synthesized a series of quinoline Schiff bases and studied their charge transfer (CT) donor/acceptor centers using TD-DFT simulations. The optical properties of the synthesized imino quinolines **5** were investigated experimentally, using photoluminescence and absorption spectroscopy. The unique HOMO-LUMO structure, reasonable quantum yield, and presence of acceptor sites in the imine molecule prompts us to embed the imino quinoline on the surface of the ZnO nanoparticles dispersed in toluene. The solution mixture was subjected to optical pumping, and a random laser was obtained. It is pertinent to note that, to the best of our knowledge, this could be the first report on a random laser achieved employing imino quinoline, embedded on ZnO nanoparticles, through pulsed laser excitation in the nanosecond range.

## 2. Results and Discussion

### 2.1. Synthesis

**Scheme 1:**
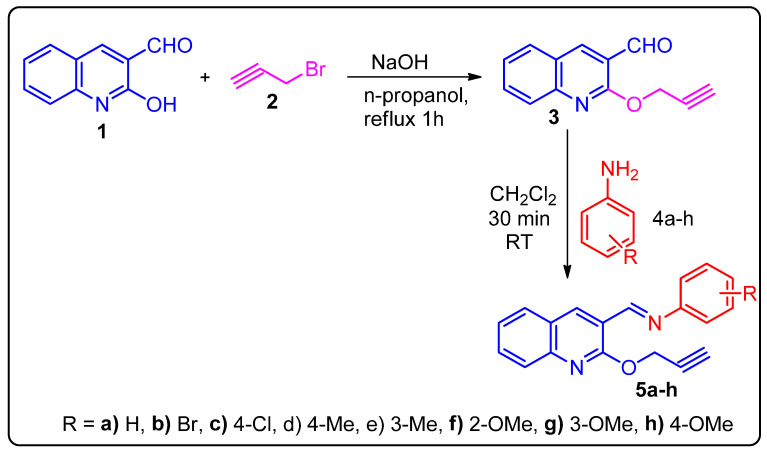
Synthesis of quinoline Schiff base, **5a**–**h**.

The required compound, (*E*)-*N*-benzylidene-2-(prop-2-yn-1-yloxy)quinolin-3-amine, **4,** was synthesized from 2-hydroxyquinoline-3-carbaldehyde **1** in two good yielding steps, as described in Scheme 1. Thus, 2-hydroxyquinoline-3-carbaldehyde **1** was *O*-propargylated in the presence of sodium hydroxide in *n*-proponal to afford 2-(prop-2-yn-1-yloxy)quinoline-3-carbaldehyde **3** in good yields. The structure of propargylated quinoline carbaldehyde was confirmed through ^1^H and ^13^C NMR spectroscopic data (*vide* Supplementary Materials). The quinoline carbaldehyde **3,** when treated with various substituted arylamines in CH_2_Cl_2_, afforded the corresponding quinoline arylimines in excellent yield (89–95%). Thus, an equimolar mixture propargylated quinoline carbaldehyde **3** and various substituted anilines **4a**–**h** in dry CH_2_Cl_2_ stirred for 30 min at room temperature. The completion of the reaction was evidenced by TLC analysis, and the solvent was removed under reduced pressure. The crude material was further washed with diethyl ether to afford pure imine **5** in good yields. The synthesis of quinoline Schiff base (**5a**–**h**) is given in Scheme 1.

**Figure 1:**
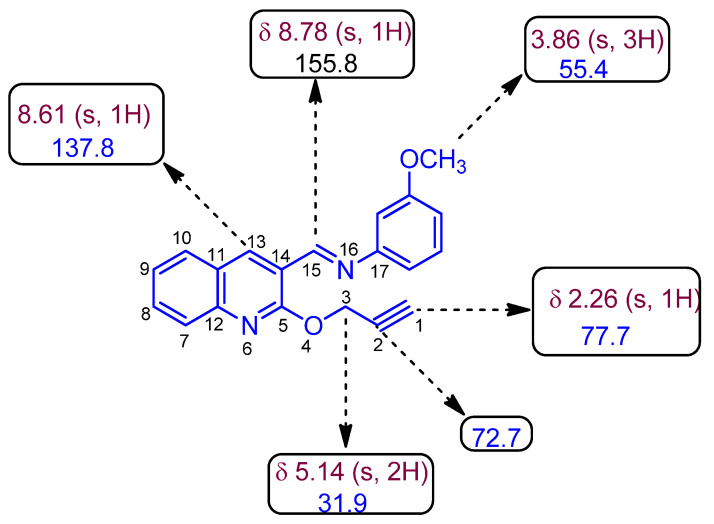
Selected chemical shift of **5g**.

**Figure 2:**
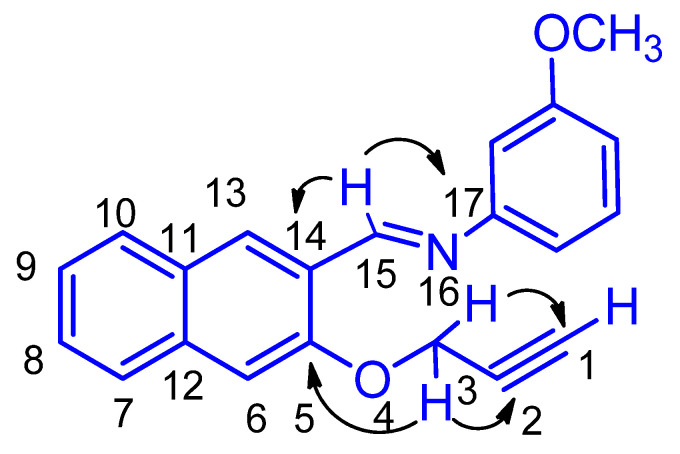
Selected HMBC of **5g**.

The structure of compound **5** agreed well with the one- and two- dimensional spectroscopic analysis, as evidenced for a representative example **5g** (Figure 1). In the ^1^H NMR spectrum of **5g**, a singlet at δ 2.26 ppm was assigned to terminal propargyl hydrogen (H-1). The signal at δ 5.14 ppm as a singlet was ascribable to methylene hydrogens (H-3) and, in addition, H-3 hydrogens showed: (i) H, H-COSY correlations to each other; (ii) HMBCs with carbon signal (C-1) and (C-5) at δ 77.7 and 160.4 ppm. A singlet at δ 8.78 ppm and δ 8.61 belonged to imine (H-15) and quinonline (H-13) peripheral hydrogens, respectively, as shown in (Figure 2). The methoxy group hydrogens appeared at δ 3.86 ppm as a singlet. The aromatic hydrogens appeared as multiplets in the region at δ 6.21 to 7.71 ppm. The imine and methoxy carbons appeared at δ 115.8 and 55.8, respectively. The carbon signal at δ 31.9 was ascribable to methylene carbon. Similarly, the carbon signal at δ 72.7 ppm was assigned to C-2 carbon. The quinoline C-13 carbon appeared at δ 137.8 ppm. All the carbon signals were identified through HMQC spectroscopic analysis.

### 2.2. Computational Studies of ***5d***

**Figure 3:**
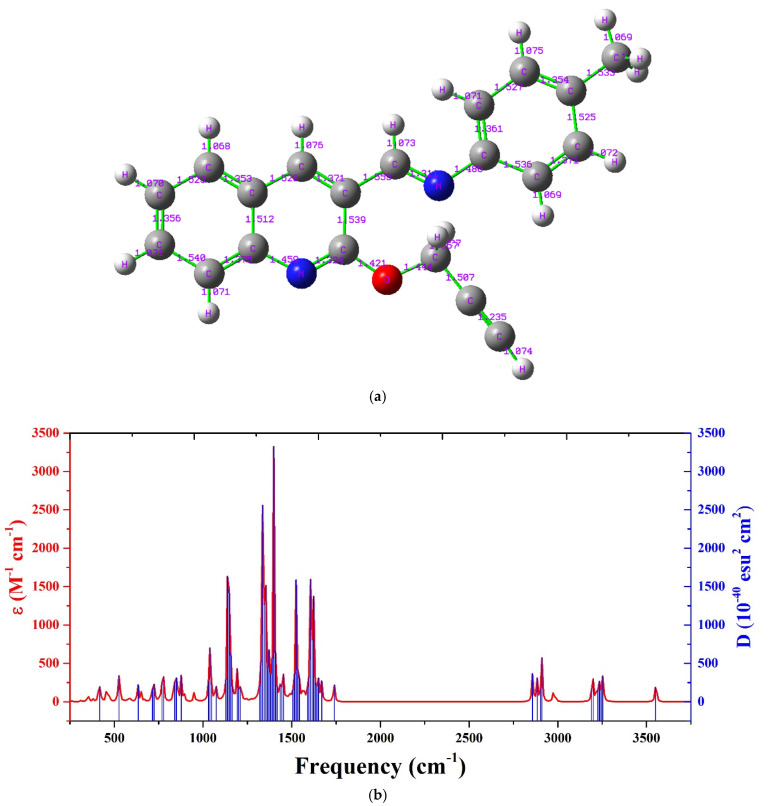
(**a**) Geometry-optimized structure of compound **5d** using the TD-DFT B3LYP method with 6–31G* basis set; (**b**) simulated IR spectra of **5d** using TD-DFT B3LYP method with 6–31G* basis set. (**a**) shows the optimized structure and (**b**) shows the infra-red (IR) spectra of **5d**.

**5d** is a linear molecule without a significant twist in the core structure, like all molecules of the synthesized series as shown Figure 3a. Figure 3b illustrates the IR spectra of **5d**, it shows major contributions of vibration from Frequency (cm^−1^) = 1036.43, 1219.41, 1399.62, and 1621.52, due to stretching of the core ring structures. Similarly, the peaks at 2909.43286, 3200.14777, and 3553.61139 were due to the stretching of peripheral hydron atoms.

**Figure 4:**
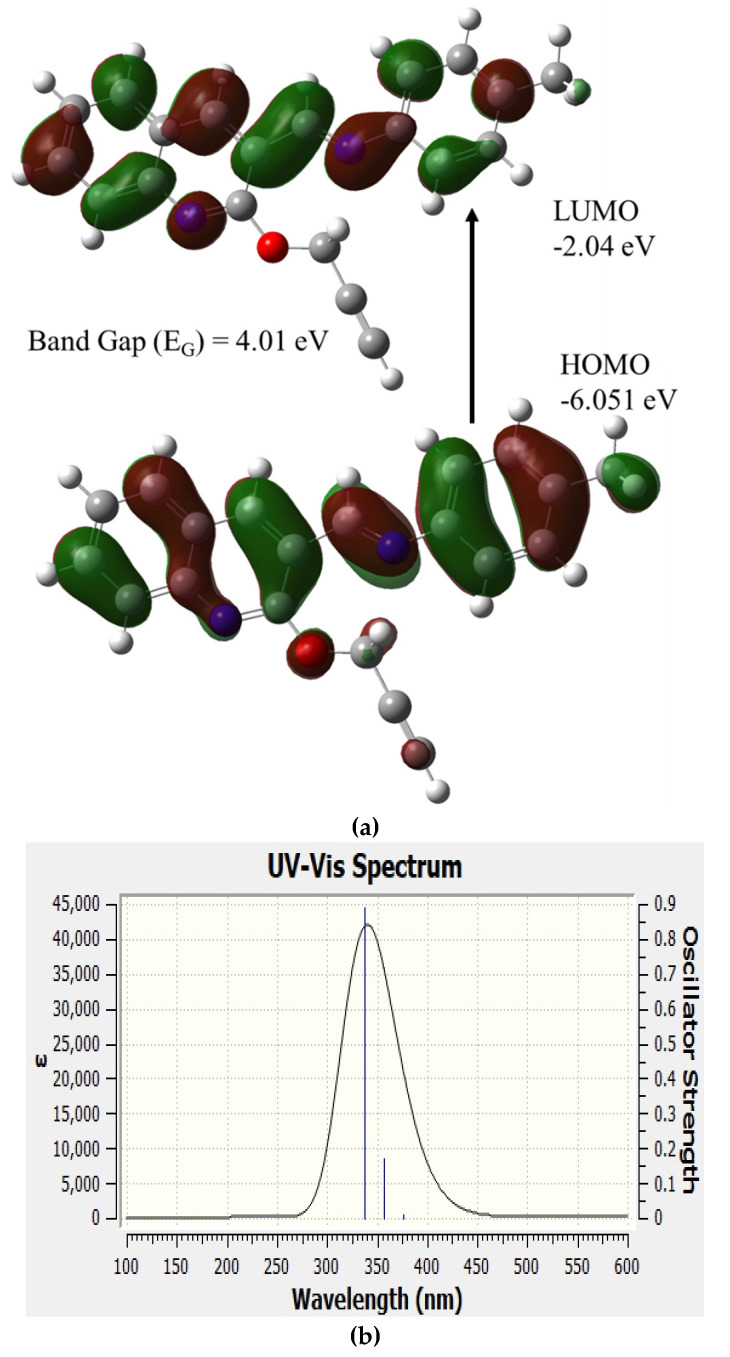

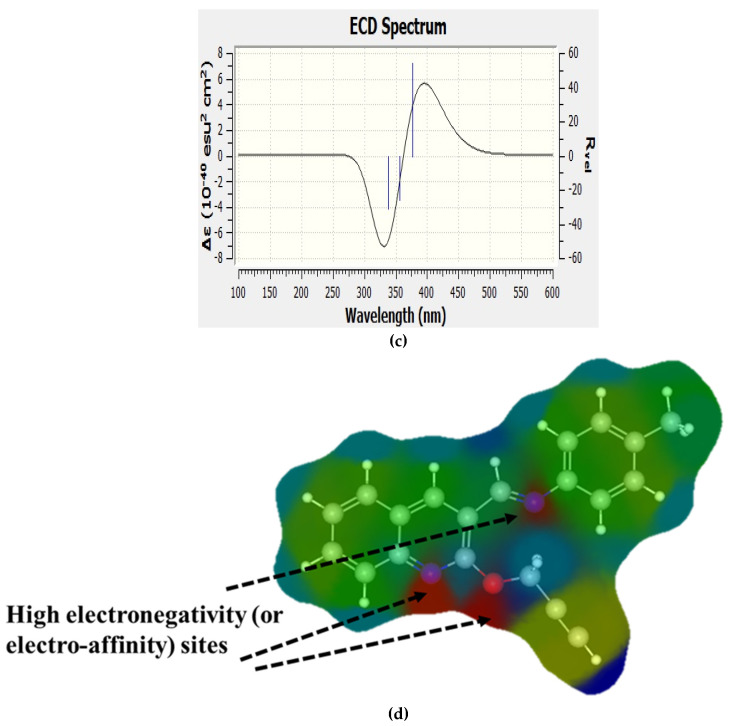
(**a**) Molecular orbital structure of **5d**, simulated using the TD-DFT B3LYP method with 6–31G* basis set; (**b**) simulated US-VIS; (**c**) ECD spectra of **5d** using TD-DFT B3LYP method with 6–31G* basis set; (**d**) calculation of charge distribution of **5d** in reverse rainbow scale.

The molecular orbital structure of **5d** is shown in Figure 4a, and electron distributions of HOMO are homogenously localized vertically in the ring structures. However, the LUMO was split into two, with major MOs present on top of the molecular structure. The HOMO and LUMO were present at −6.051 eV and −2.04 eV, with an energy gap of 4.01 eV. The HOMO and LUMO gap had contributions from three singlet oscillations, namely S_0_–S_1_, S_0_–S_2_, and S_0_–S_3_ vibrational bands at 3.2952 eV (376.26 nm), 3.4785 eV (356.43 nm), and 3.6723 eV (337.62 nm) with an oscillator strength of 0.0121, 0.1700 and 0.8922, respectively. This shows that the unique optical properties of imine compounds arise from a large bandgap. This also shows they are fluorescent molecules with singlet oscillation, and have no charge trapping triplet states. The total energy of compound 5c was −955.98 kJ mol^−1^. The total dipole moment was 0.1228 **e·nm**, indicating it is a low to moderately polar molecule. Therefore, it could easily dissolve in non-polar solvents, such as tetrahydrofuran, toluene, and chloroform.

The simulated UV-visible spectra is displayed in Figure 4b; the spectra showed three oscillator strengths at 3.2952 eV (376.26 nm), 3.4785 eV (356.43 nm) and 3.6723 eV (337.62 nm) with an amplitude of 0.0121, 0.1700 and 0.8922, respectively. The peak was at 337.62 nm. The ECD spectra of **5d** is shown in Figure 4c, and represents the variation in absorption of right and left circularly polarized light related to the electronic transitions of **5d** chromophore. The ECD showed a negative cotton effect below 356.43 nm, with a peak Δε (10^−40^ esu^2^ cm^2^) value of −7.13 and a positive cotton effect above 356.43 nm, with a peak Δε value of 5.58, indicating a strong interaction between the photons and molecules.

Figure 4d illustrates the simulated acceptor sites and charge distribution of the **5d** in reverse rainbow scale to clearly show the spatial distribution of the acceptor site in 3D space; the simulations show that **5d** has four acceptor sites and three acceptor counts, all present in nitrogen and oxygen atoms in the molecule. The presence of acceptor sites indicates that **5d** would be attached to a nanoparticle rich in electron-donating capacity.

### 2.3. Experimental Study of Steady-State Optical Properties

**Figure 5:**
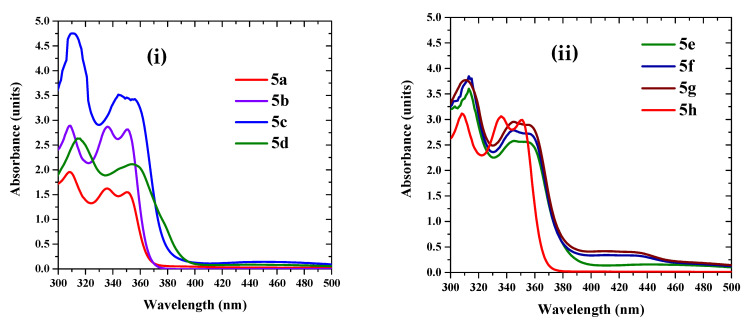

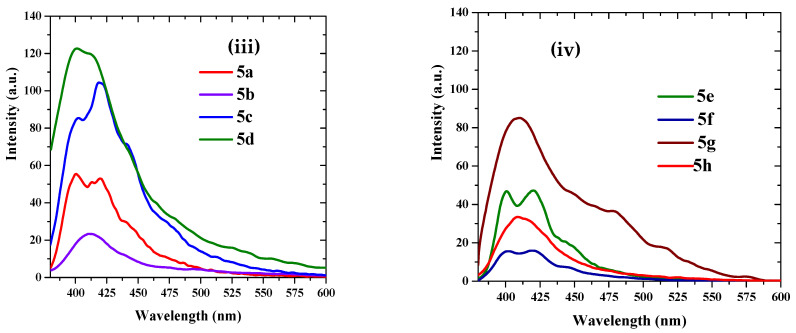
(**i**,**ii**) the absorption spectra of **5a**–**d** and **5e**–**h,** respectively; (**iii**,**iv**) the fluorescence spectra of **5a**–**d** and **5e**–**h**, respectively.

We investigated steady-state optical properties, such as absorption and fluorescence, of all the compounds synthesized in this series. The compounds were dissolved in various organic solvents to form different concentrations. The investigations showed that, of all solvents, toluene gave better fluorescence and quantum yield (Q.Y.). To keep the report concise, we skipped the detailing of solvent effects and concentration. The Figure 5i,ii shows the absorption spectra of compounds 1–4 and compounds 5–8, respectively, for a concentration of 0.5 mg of 1 mL of toluene. The profile of absorption spectra showed either three peaks or two peaks; the peak details are given in Table 1. In comparison with simulated UV-VIS spectra, the peaks around 310 nm, 335 nm, and 360 nm corresponded to the vibrational transitions S_0_–S_3_, S_0_–S_2_, and S_0_–S_1_. The variation between simulated and experimental spectra, with respect to oscillator strength, was around 15 nm, which could be attributed to experimental concentrations. Similarly, Figure 5iii, and iv show the fluorescence spectra of Imm 1–4 and 5–8, respectively. The fluorescence spectra had three peaks, or two peaks and one shoulder, and the peaks could be attributed to the vibrational transition described above. The peak of absorption and fluorescence, Stokes shift (Δλ), and quantum yield (Q.Y.) were calculated and given in Table 1. The quantum yield was calculated using Equation (1) [26,27], and the Rhodamine 101 was used as the reference with the Q.Y. of unity [28].

(1) $$Q_{s\;}=\frac{\sum I_{S}}{\sum I_{R}}\times \frac{\;OD_{r}}{OD_{s}}\times \left(\frac{n_{s}}{n_{r}}\right){\;}^{2}\times Q_{r}$$

where $Q_{s\;}$ and $Q_{r}$ represents the Q.Y. of the sample and reference, $\sum I_{S}$ and $\sum I_{R}$ represent the integral area under normalized fluorescence [i.e., $\sum I_{s}=\int I_{s}\cdot \left(\bar{\nu }\right)\;d\bar{\nu }$, and $\sum I_{R}=\int I_{R}\cdot \left(\bar{\nu }\right)\;d\bar{\nu }$], here $\bar{\nu }$ is frequency corresponding to the wavelength. OD is the optical density at the excitation wavelength (355 nm), and n is the refractive index of the respective solvent. The r indicated the reference, and s indicated the sample.

### 2.4. Random Laser Studies

**Figure 6:**
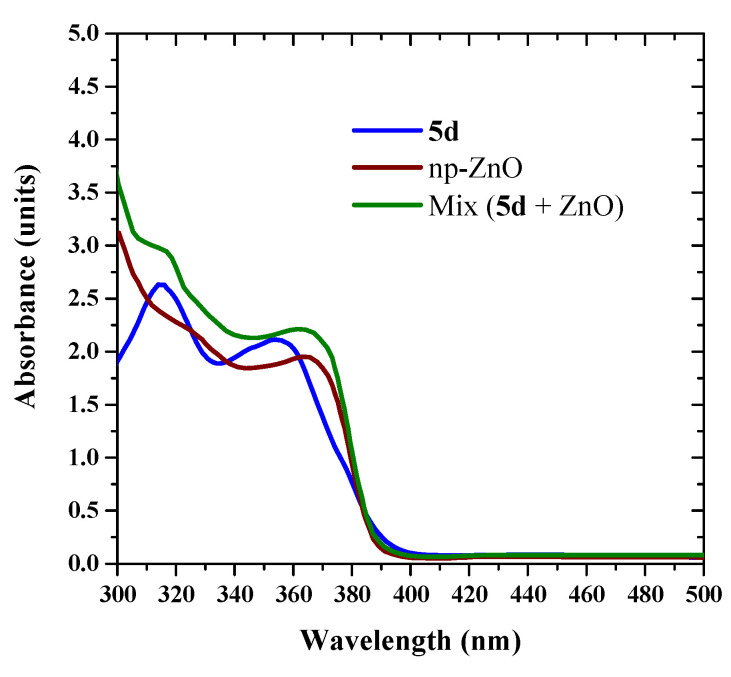
The absorption spectra of **5d**, ZnO and the mix solution in toluene.

Compound **5d** had the best quantum yield of all synthesized compounds, the calculated Q.Y. of **5d** was 0.3, and the Stokes was 60 nm, as shown in Table 1. Therefore, the compound **5d** has the best chance of producing a random laser. Hence, a random laser was designed using ZnO nanoparticles of an average size of 90 nm. The shape of synthesized ZnO resembled flakes. The ZnO of concentration 5 mg/mL was dispersed into toluene, to form solution 1 (Solution 1). The concentration of compound 5 was dispersed in 1 mg/mL of the same solvent to form solution 2 (Solution 2). The mixture solution was formed by adding both solutions (Solution 1 and Solution 2) in a 1:1 *v*/*v* ratio. Due to the presence of the acceptor sites in compound **5d**, it attached to the ZnO nanoparticle. Figure 6 shows the absorption spectra of the mix solution in comparison with pure ZnO nanoparticles and pure compound **5d** in toluene, for above said concentrations. The absorption spectra showed an excellent match between ZnO and compound **5d** absorption spectra, and the mixing improved the absorption slightly. The fluorescence of ZnO nanoparticles was weak, which is associated with the defecting state fluorescence of ZnO [29].

Both ZnO and compound **5d** pure did not produce any laser action for any given pump energies at 355 nm, but they did produce laser-induced fluorescence (LIF). On the other hand, the mixed solution was active for high-energy optical pumping produced reasonable LIF. When the pump energy was below 15 ± 1 mJ, the mixture produced LIF without sharp peaks.

**Figure 7:**
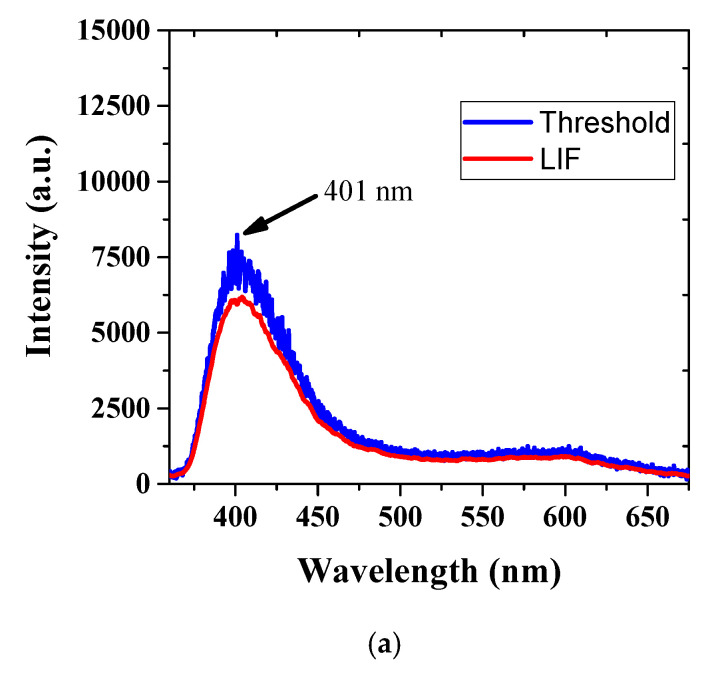

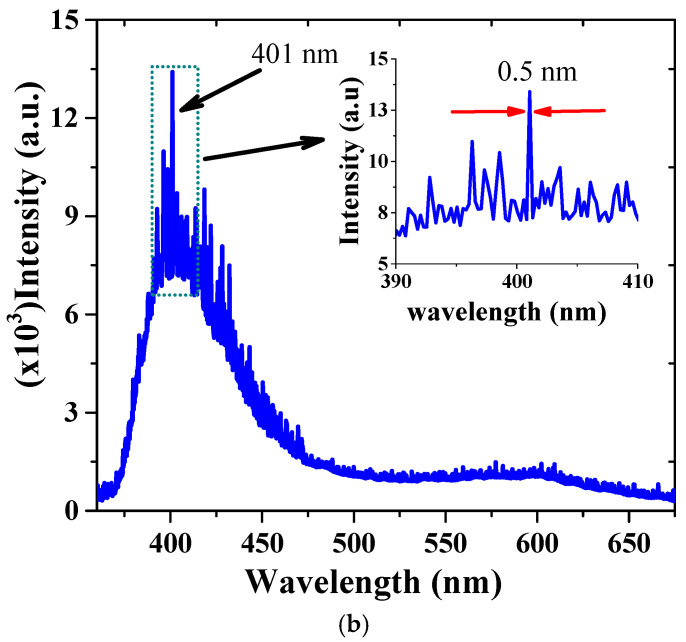
(**a**) the threshold and LIF spectra of compound **5d** + ZnO mixer solution in toluene; (**b**) the random laser spectra of **5d** + ZnO mixer solution in toluene, inset the zoom of same for wavelength range from 390 nm to 410 nm, shows the random laser peak at 401 nm with a sharp 0.5 nm FWHM.

When the pump energy was raised above the threshold (17 mJ), the sharp peaks started to appear, as shown in Figure 7a (blue line). With the further increase in pump energy (20 mJ) the sharp peaks grew, as shown in Figure 7b. The inset of Figure 7b shows the spikes were very sharp, and the full width at half the maximum was 0.5 nm. Since the optical gain was random, the peak emission wavelength changed. It is important to note compound **5d** did not lase without ZnO, but any nanoparticle such as SiO_2_, TiO_2_ or Al_2_O_3_ can also be used as a core to produce the random loop optical gain. The nanosized and random aggregation and arrangement of the particles enabled a random gain due to the random scattering on the surface of the nanoparticle, meaning the loop may have involved one, or many, nanoparticles. Nevertheless, this random laser from the novel compound **5d** could be useful in the design of tunable laser with low cost. If the same mixture is embedded into a polymer matrix, it could be good candidate for a solid-state tunable laser.

## 3. Materials and Methods

### 3.1. Synthesis of Propargylated Quinoline Carbaldehyde, ***3***

The 2-hydroxyquinoline-3-carbaldehyde (1 mmol) was dissolved in ethanol, followed by the addition of NaOH solution (1 mmol). The reaction mixture was stirred for 10 min, and then propargyl bromide was added slowly to the reaction mixture. The completion of reaction was evidenced by thin layer chromatography (TLC), and the solvent was removed under reduced pressure. The residue was purified by column chromatography.

2-(prop-2-yn-1-yloxy)quinoline-3-carbaldehyde: **3** white solid; ^1^H NMR 2.27–2.28 (m, 1H), 5.12–5.13 (m, 2H), 7.30–7.34 (m, 1H, ArH), 7.53–7.55 (m, 1H, ArH), 7.71–7.75 (m, 2H, ArH), 8.37 (s, 1H, ArH), 10.44 (s, 1H, CHO); ^13^C NMR: 31.6, 73.0, 76.9, 115.1, 119.6, 123.6, 125.2, 132.1, 134.1, 140.8, 141.8, 161.0, 189.87; Mass: *m/z*: = 211 (M^+^), Anal. Calcd for C_13_H_9_NO_2_; C, 73.92; H, 4.29; N, 6.63; Found C, 73.84; H, 4.19; N, 6.74%.

### 3.2. General Synthetic Procedure for (E)-N-benzylidene-2-(prop-2-yn-1-yloxy)quinolin-3-amine, ***5a–h***

A mixture of 2-(prop-2-yn-1-yloxy)quinoline-3-carbaldehyde **3** (1 mmol) and appropriate arylamine **4** (1 mmol) in dry CH_2_Cl_2_ was stirred at room temperature for 30 min. After completion of the reaction, as confirmed by thin layer chromatography (TLC), the solvent was evaporated under reduced pressure. The crude imine was washed with diethyl ether to obtain pure imine in good yields.

(E)-*N*-(benzylidene)-2-(prop-2-yn-1-yloxy)quinolin-3-amine:**5a** Pale yellow solid; ^1^H NMR 2.28 (s, 1H), 5.17–5.18 (m, 2H), 7.24–7.34 (m, 4H, ArH), 7.39–7.43 (m, 2H, ArH), 7.54–7.57 (m, 1H, ArH), 7.65–7.70 (m, 1H, ArH), 7.74–7.76 (m, 1H, ArH), 8.66 (s, 1H, ArH), 8.97 (s, 1H, CH=N); ^13^C NMR: 31.8, 51.8, 72.6, 77.7, 114.7, 120.5, 121.3, 123.2, 126.4, 126.5, 126.7, 129.2, 130.7, 132.1, 137.6, 139.4, 151.6, 155.6, 161.1. Mass: *m*/*z*: = 286 (M^+^), Anal. Calcd for C, 79.70; H, 4.93; N, 9.78; Found C, 79.78; H, 5.02; N, 9.89%.

(E)-*N*-(4-bromobenzylidene)-2-(prop-2-yn-1-yloxy)quinolin-3-amine:**5b** Pale yellow solid; ^1^H NMR 2.26 (s, 1H), 5.14–5.15 (m, 2H), 7.10–7.14 (m, 2H, ArH), 7.28–7.32 (m, 1H, ArH), 7.46–7.55 (m, 3H, ArH), 7.64–7.68 (m, 1H, ArH), 7.71–7.75 (m, 1H, ArH), 8.62 (s, 1H, ArH), 8.91 (s, 1H, CH=N); ^13^C NMR: 36.2, 77.0, 81.8, 119.1, 124.3, 124.7, 127.2, 127.5, 130.4, 135.1, 136.6, 142.2, 143.8, 154.8, 160.3, 165.3. Mass: *m*/*z*: = 365 (M^+^), Anal. Calcd for C_19_H_13_BrN_2_O: C, 62.48; H, 3.59; N, 7.67; Found C, 62.59; H, 3.70; N, 7.75%.

(E)-*N*-(4-chlorbenzylidene)-2-(prop-2-yn-1-yloxy)quinolin-3-amine:**5c** Pale yellow solid; ^1^H NMR 2.24–2.26 (m, 1H), 5.15–5.16 (m, 2H), 7.22–7.31 (m, 3H, ArH), 7.36-7.40 (m, 2H, ArH), 7.52–7.54 (m, 1H, ArH), 7.63–7.67 (m, 1H, ArH), 7.72–7.73 (m, 1H, ArH), 8.64 (s, 1H, ArH), 8.94 (s, 1H, CH=N); ^13^C NMR: 35.5, 55.4, 74.7, 76.2, 110.1, 118.3, 124.1, 124.9, 126.8, 130.0, 130.1, 132.8, 130.7, 134.3, 135.8, 141.2, 155.2, 164.7. Mass: *m*/*z*: = 320 (M^+^), Anal. Calcd for C_19_H_13_ClN_2_O: C, 71.14; H, 4.08; Cl, 11.05; N, 8.73; Found C, 71.25; H, 4.17; N, 8.84%.

(E)-*N*-(4-methylbenzylidene)-2-(prop-2-yn-1-yloxy)quinolin-3-amine: **5d** Pale yellow solid; ^1^H NMR 2.26–2.27 (m, 1H), 3.37 (s, 3H), 5.15–5.16 (m, 2H), 7.20–7.25 (m, 5H, ArH), 7.30 (t, *J* = 7.5 Hz, 1H, ArH), 7.53 (d, *J* = 8.5 Hz, 1H, ArH), 7.65 (t, *J* = 7.5 Hz, 1H, ArH), 7.73 (d, *J* = 7.5 Hz, 1H, ArH), 8.63 (s, 1H, ArH), 8.96 (s, 1H, CH=N); ^13^C NMR: 21.2, 31.9, 72.7, 77.8, 114.8, 120.6, 121.3, 123.2, 126.6, 129.9, 130.7, 132.0, 136.6, 137.4, 139.5, 149.0, 154.6, 161.2. Mass: *m*/*z*: = 300 (M^+^), Anal. Calcd for C_20_H_16_N_2_O: C, 79.98; H, 5.37; N, 9.33; Found C, 80.04; H, 5.48; N, 9.45%.

(E)-*N*-(3-methylbenzylidene)-2-(prop-2-yn-1-yloxy)quinolin-3-amine: **5e** Pale yellow solid; ^1^H NMR 2.27–2.28 (m, 1H), 3.38 (s, 3H), 5.17–5.18 (m, 2H), 7.21–7.35 (m, 5H, ArH), 7.33 (t, *J* = 7.5 Hz, 1H, ArH), 7.56 (d, *J* = 8.5 Hz, 1H, ArH), 7.66 (t, *J* = 7.5 Hz, 1H, ArH), 7.75 (d, *J* = 7.5 Hz, 1H, ArH), 8.65 (s, 1H, ArH), 8.98 (s, 1H, CH=N); ^13^C NMR: 21.3, 32.1, 72.9, 78.0, 114.9, 120.8, 121.5, 123.4, 126.8, 130.1, 130.8, 132.2, 132.4, 136.5, 136.8, 137.6, 139.6, 149.2, 154.8, 161.4. Mass: *m/z*: = 300 (M^+^), Anal. Calcd for C_20_H_16_N_2_O: C, 79.98; H, 5.37; N, 9.33; Found C, 80.05; H, 5.45; N, 9.42%.

(E)-*N*-(2-methoxybenzylidene)-2-(prop-2-yn-1-yloxy)quinolin-3-amine: **5f** Pale yellow solid; ^1^H NMR 2.26 (s, 1H), 3.82 (s, 3H), 5.14–5.15 (m, 2H), 6.69–6.78 (m. 4H, ArH), 6.94–7.06 (m, 1H, ArH), 7.19–7.30 (m, 1H, ArH), 7.50–7.52 (m 1H, ArH), 7.61–7.63 (, 1H, ArH), 7.70–7.72 (m, 1H, ArH), 8.69 (s, 1H, ArH), 8.96 (s, 1H, CH=N); ^13^C NMR: 31.7, 55.3, 72.6, 77.7, 110.3, 114.9, 118.4, 120.4, 120.9, 126.4, 127.3, 136.1, 137.8, 139.4, 141.3, 147.2, 152.5, 156.3, 161.0. Mass: *m*/*z*: = 316 (M^+^), Anal. Calcd for C_20_H_16_N_2_O_2_: C, 75.93; H, 5.10; N, 8.86; Found C, 76.03; H, 5.18; N, 8.95%.

(E)-*N*-(3-methoxybenzylidene)-2-(prop-2-yn-1-yloxy)quinolin-3-amine: **5g** Pale yellow solid; ^1^H NMR 2.26 (s, 1H), 3.86 (s, 3H), 5.14 (s, 2H), 6.21–6.29 (m, 1H, ArH), 6.78–6.85 (m, 3H, ArH), 7.23–7.30 (m, 2H, ArH), 7.50–7.52 (m, 1H, ArH), 7.62–7.65 (m, 1H, ArH), 7.70–7.71 (m, 1H, ArH), 8.61 (s, 1H, ArH), 8.93 (s, 1H, CH=N); ^13^C NMR: 31.9, 55.4, 72.7, 77.7, 106.7, 112.7, 113.5, 114.8, 120.5, 123.3, 126.4, 130.0, 130.8, 132.2, 137.8, 139.5, 153.0, 155.8, 160.4; Mass: *m*/*z*: = 316 (M^+^), Anal. Calcd for C_20_H_16_N_2_O_2_: C, 75.93; H, 5.10; N, 8.86; Found C, 76.05; H, 5.20; N, 8.98%.

(E)-*N*-(4-methoxybenzylidene)-2-(prop-2-yn-1-yloxy)quinolin-3-amine: **5h** Pale yellow solid; ^1^H NMR 2.25–2.26 (m, 1H), 3.83 (s, 3H), 5.14–5.15 (m, 2H), 6.91–6.94 (m, 2H, ArH), 7.24–7.32 (m, H, ArH), 7.52 (d, J = 8.0 Hz, 1H, ArH), 7.61–7.64 (m, 1H, ArH), 7.72 (d, *J* = 8.4 Hz, 1H, ArH), 8.62 (s, 1H, ArH), 8.97 (s, 1H, CH=N); ^13^C NMR: 31.8, 55.5, 72.6, 77.7, 114.4, 114.6, 120.5, 122.7, 123.1, 126.6, 130.6, 131.8, 136.9, 139.2, 144.3, 153.1, 158.7, 161.2. Mass: *m*/*z*: = 316 (M^+^), Anal. Calcd for C_20_H_16_N_2_O_2_: C, 75.93; H, 5.10; N, 8.86; Found C, 76.01; H, 5.21; N, 8.93%.

### 3.3. Spectral Studies

**Figure 8:**
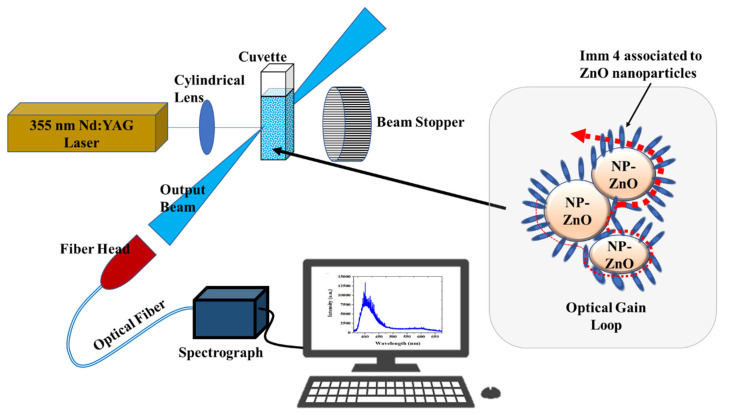
Experimental set-up; (inset) schematic of **5d** attached to ZnO nanoparticle and random laser gain loop.

The synthesis of (E)-4-methyl-N-((2-(prop-2-yn-1-yloxy)quinolin-3-yl)methylene)aniline from propagated quinoline carbaldehyde, **3**, had a molecular weight (Mw) of 300.361 g mol^−1^. The ZnO nanoparticles were synthesized using an opti-sense nanoparticle synthesizer through the hydrothermal method, as reported before [30]. Its average size distribution was 100 nm, with particle size ranging from 30–180 nm. A Perkin–Elmer Lambda 950 spectrophotometer (Llantrisant, United Kingdom) was used to record the absorption spectra of the film samples in the range of 100 to 1100 nm. The fluorescence spectra at the excitation wavelength of 300–360 nm (depending on excitation peak) from a xenon flash lamp was measured using a spectrofluorometer (LS 55, from the same company). The laser experimental set-up and formation of the ZnO- **5d** random optical gain loop is depicted in Figure 8 (modified version of ref. [31,32]). To achieve transverse pumping, the third harmonic (355 nm) of a 5 ns Nd:YAG laser was focused by a quartz cylindrical lens (5 cm focal length). The cylindrical lens focused the pulse onto a horizontal strip of 1 cm × 2 mm (length × width). The dimension of the Cuvette was 1 × 1 × 4 cm (L × B × H), and it was filled with a solution mixture of A and B, where A was 2 mg/mL of ZnO in Toluene and B was 1 mg/mL of **5d** in toluene. The output light was fed to optic fiber connected to a spectrometer (Ocean Optics Range 300–1200 nm, resolution ≤0.5 nm). The output data was acquired and recorded on a PC. A beam stopper was placed behind the Cuvette to stop residual light from the pump laser. All simulations were accomplished with Avogadro, Marvin Sketch, and Gaussian 16 program package [33] at King Saud University using a workstation equipped with AMD Ryzen 9 3950X 16-core, 32-Thread, 128 GB RAM, 1 TB M.2 SSD, with an FORTRAN emulator. The B3LYP method with basis set 6–31G* was used for all time-dependent DFT (TDDFT) and density functional theory (DFT) calculation of imino quinolines [34,35].

## 4. Conclusions

A small library of hitherto unexplored new class of conjugated (*E*)-*N*-((2-(prop-2-yn-1-yloxy)quinolin-3-yl)methylene)anilines were synthesized in two good yielding steps. The structure of the newly synthesized imines was elucidated through spectroscopic analysis. The synthesized compounds were thus investigated for their photochemical and lasing ability. The HOMO-LUMO structure, acceptor sites, and UV-VIS spectra were simulated. Compound **5d** was embedded onto ZnO nanoparticles, and optically pumped with a 355 nm, 5ns pump pulse. For pump energy less than 15 mJ, the mixture solution produced only LIF. However, at around 15 mJ, a sharp spikes appeared and grew intensively, with an increase in pump energy. The sharp spikes were spectrally very narrow (Δλ = 0.5 nm), indicating that the imino quinoline produced random lasers in the range 400–430 nm.

## Figures and Tables

**Table 1:** Measured fluorescence and absorption peak, along with calculated Stokes shift and quantum yield of synthesized compounds **5a**–**h**.

Compound	Fluorescence Peak (nm)		Absorption Peak (nm)			Stokes Shift (Δλ)	Quantum Yield $Q_{s\;}$
	S_0–1_	S_0–0_	S_0–1_	S_0–2_	S_0–3_		
5a	421	400	351	335	309	70	0.21
5b	411	-	349	335	308	62	0.19
5c	418	403	350	-	311	68	0.14
5d	420	409	360	344	314	60	0.3
5e	420	400	355	345	313	65	0.157
5f	-	401	350	-	315	51	0.18
5g	-	409	356	344	312	53	0.193
5h	-	408	351	336	308	57	0.23

## Data Availability

Data available on request.

## Supplementary Materials

The following supporting information can be downloaded, Figures S1–S4: 1H, 13C NMR spectrum of 3 and 5d; Figures S5–S10: 1H, 13C NMR, DEPT, COSY, HMQC, HMBC spectrum of 5g. Figures S11–S18: Mass spectrum of 5a–g.

## Sample Availability

Samples used in this study are available from the authors.
