# Supplementary material for: Investigation of the Optical Properties of a Novel Class of Quinoline Derivatives and Their Random Laser Properties Using ZnO Nanoparticles
Source: Molecules. 2021 Dec 27;27(1):145. doi: 10.3390/molecules27010145 (PMC8746827; doi:10.3390/molecules27010145)
Supplement: Supplementary file 1 [file molecules-27-00145-s001.zip › molecules-1496342-supplementary.pdf]

Supplementary File

Investigation of the Optical Properties of a Novel Class of Quinoline Derivatives and Their Random Laser Properties Using ZnO Nanoparticles

General method

Chemistry

General

^{1}H and ^{13}C NMR spectra were recorded on Varian Mercury JEOL-400/500 NMR spectrometers in $CDCl_3$, using TMS as the internal standard. Chemical shifts are given in parts per million (δ-scale) and coupling constants are given in hertz. Elemental analyses were performed on a Perkin–Elmer 2400 Series II Elemental CHNS analyzer. Mass spectra were recorded on a Quattro Premier™ instrument (Micromass, Milford, USA) equipped with an electrospray ionization source (Zespray), coupled with an Acquity® UPLC system.

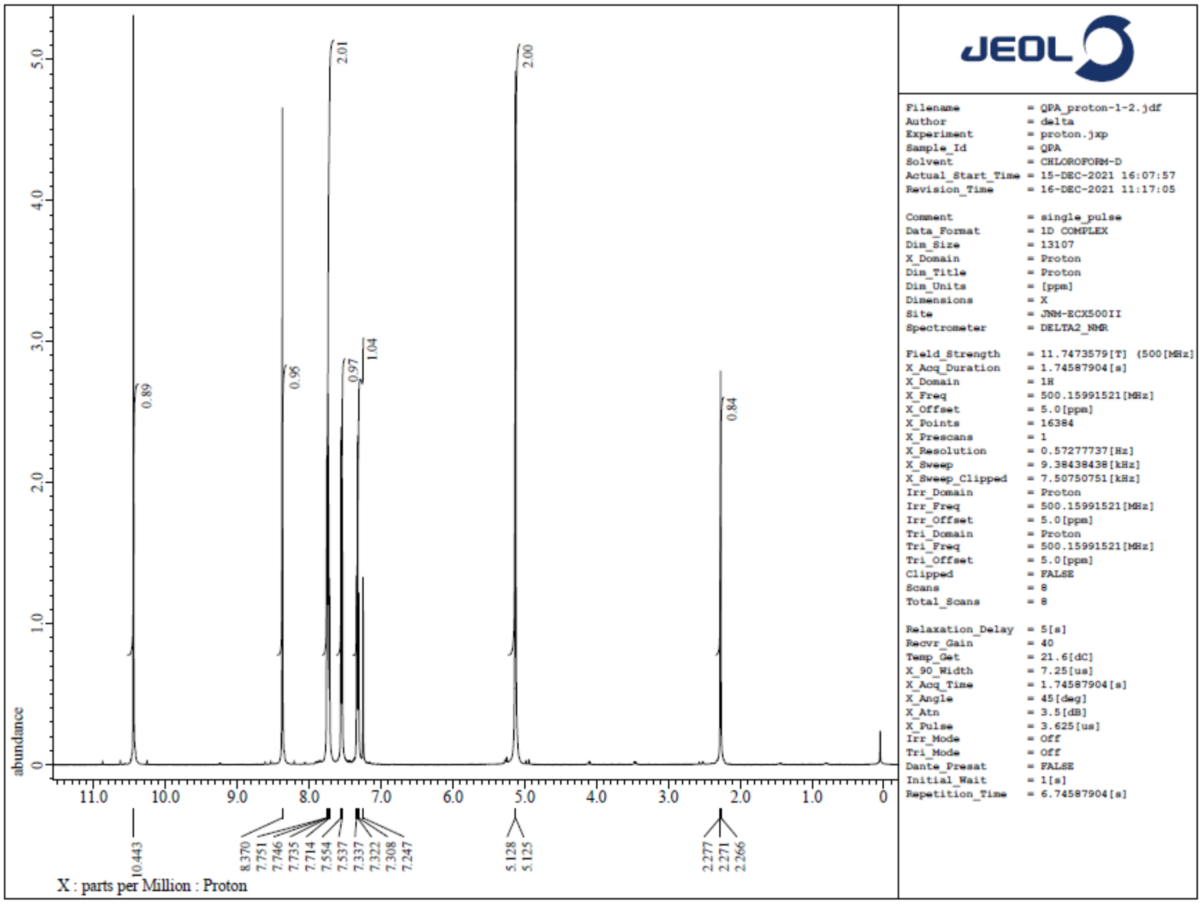

Figure S1. 1H NMR spectrum of 3

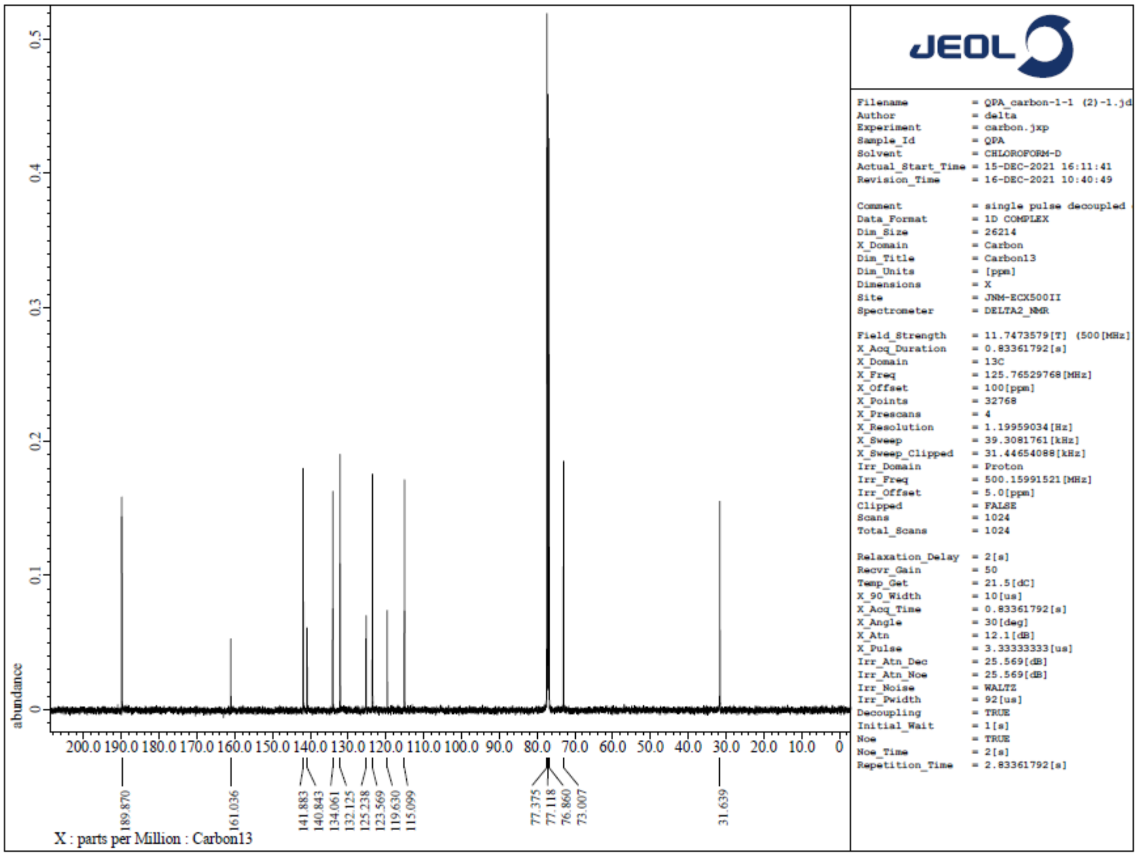

Figure S2. ^{13}C NMR spectrum of **3**

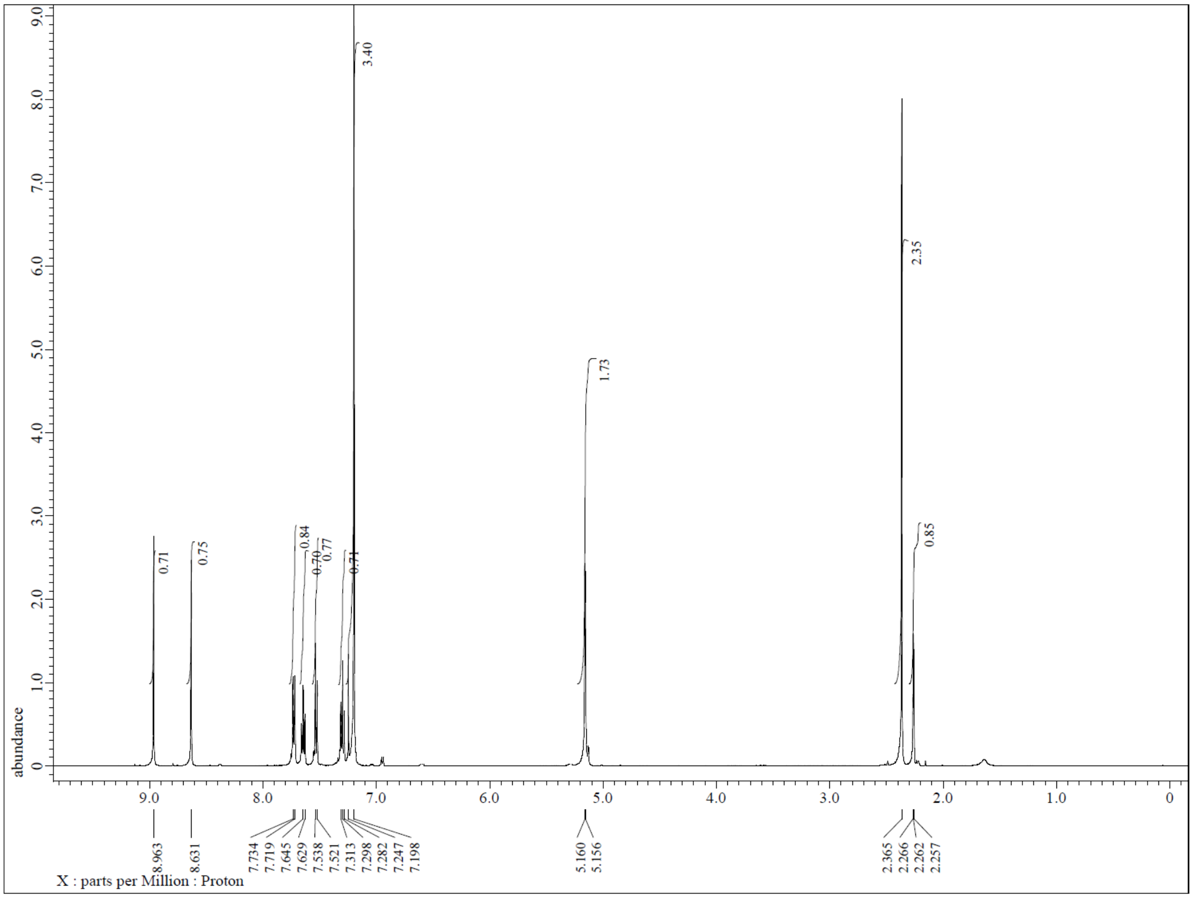

Figure S3. ^{1}H NMR spectrum of **5d**

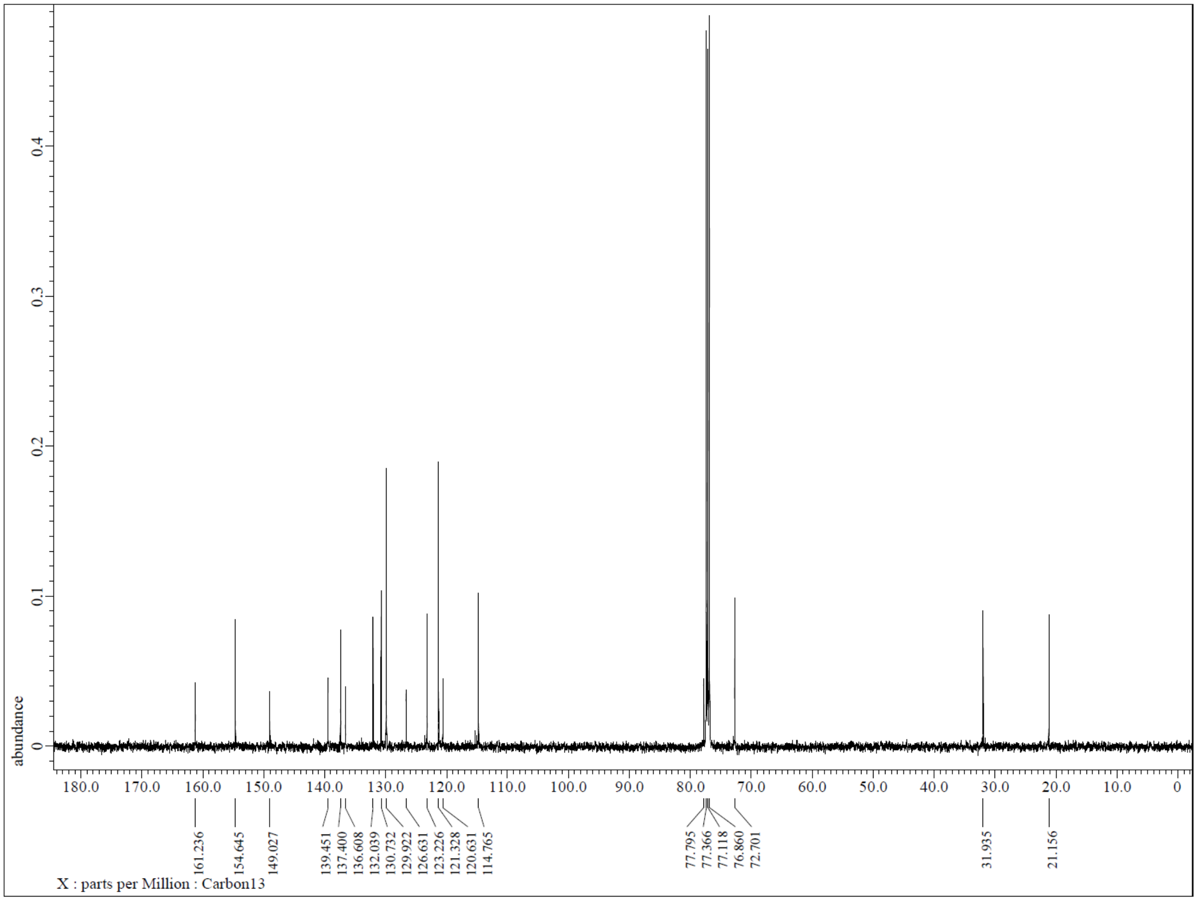

Figure S4 ^{13}C NMR spectrum of **5d**

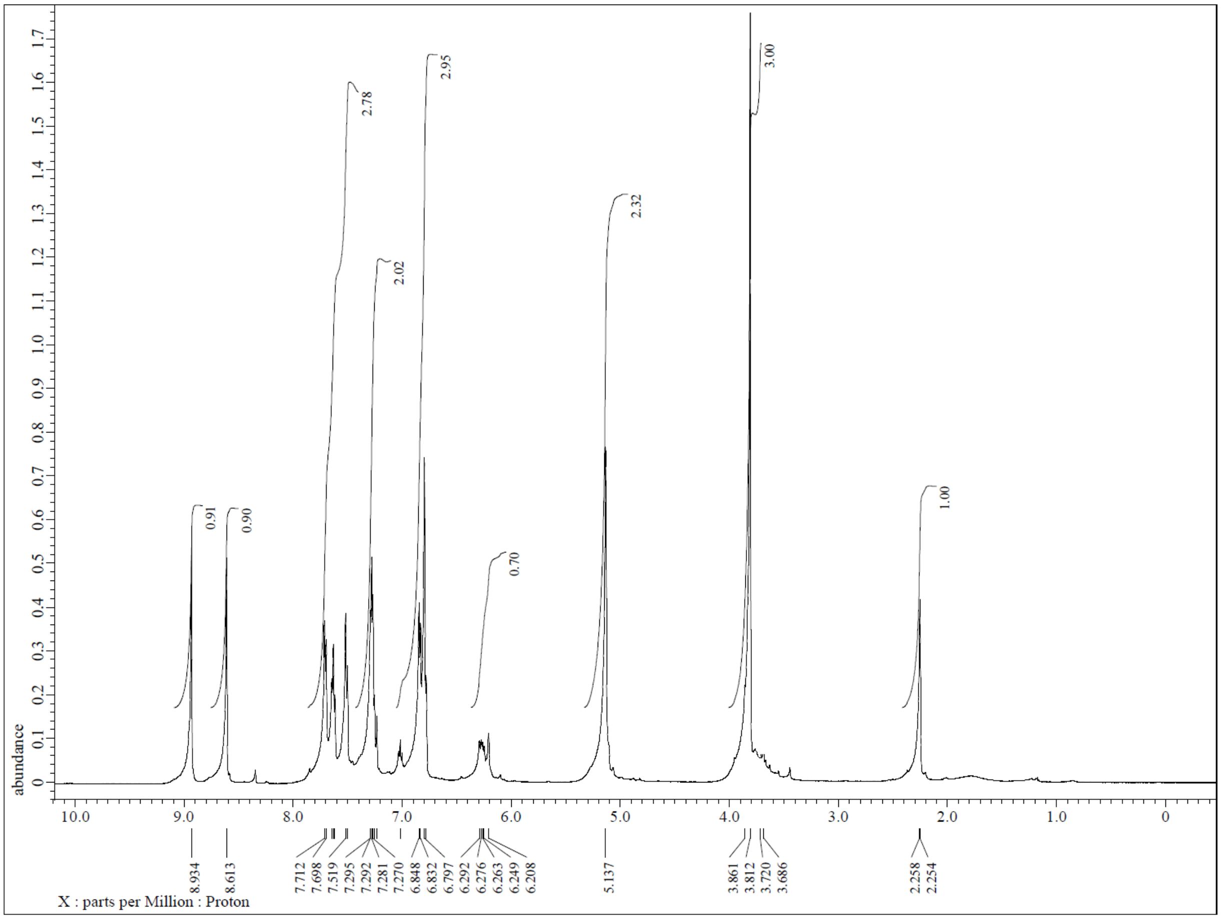

Figure S5. ^{1}H NMR spectrum of **5g**

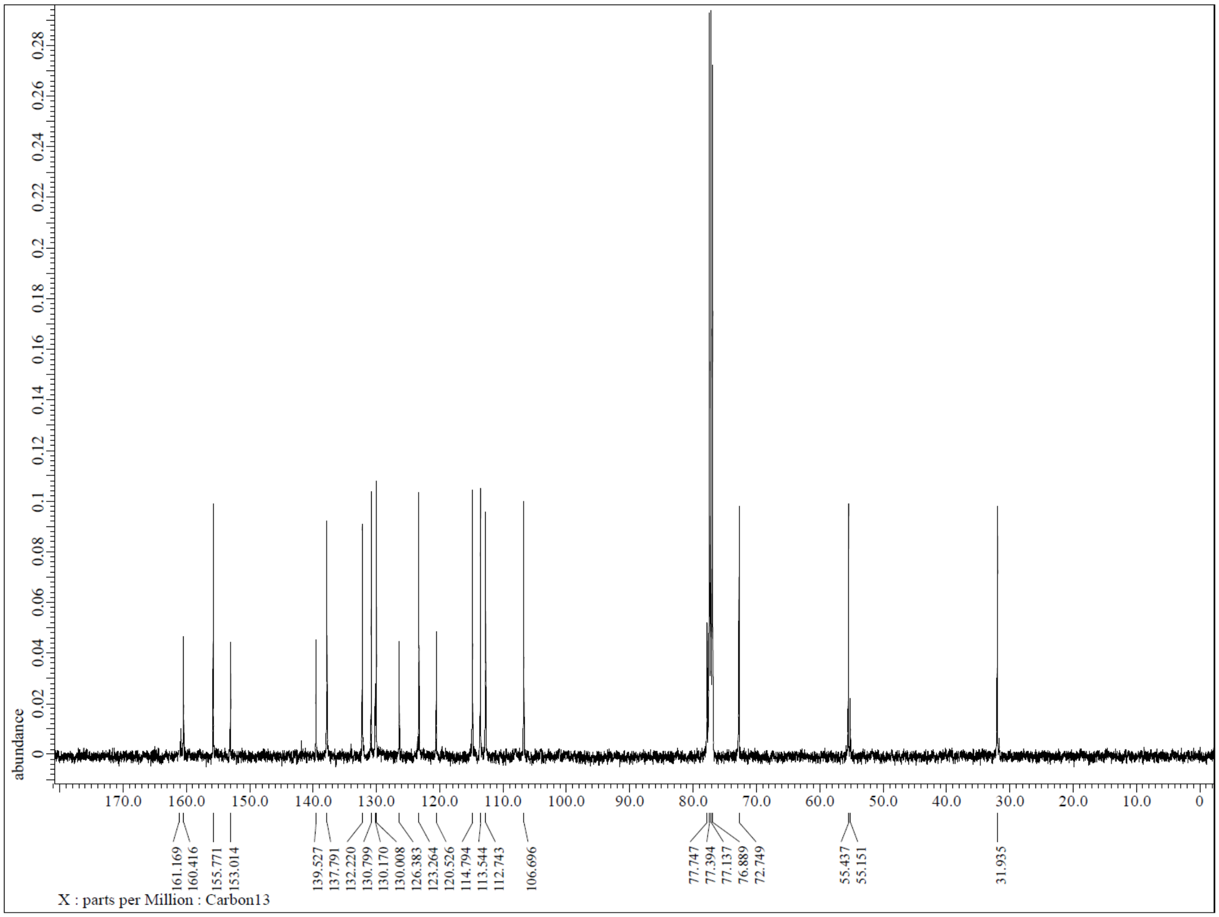

Figure S6 ^{13}C NMR spectrum of **5g**

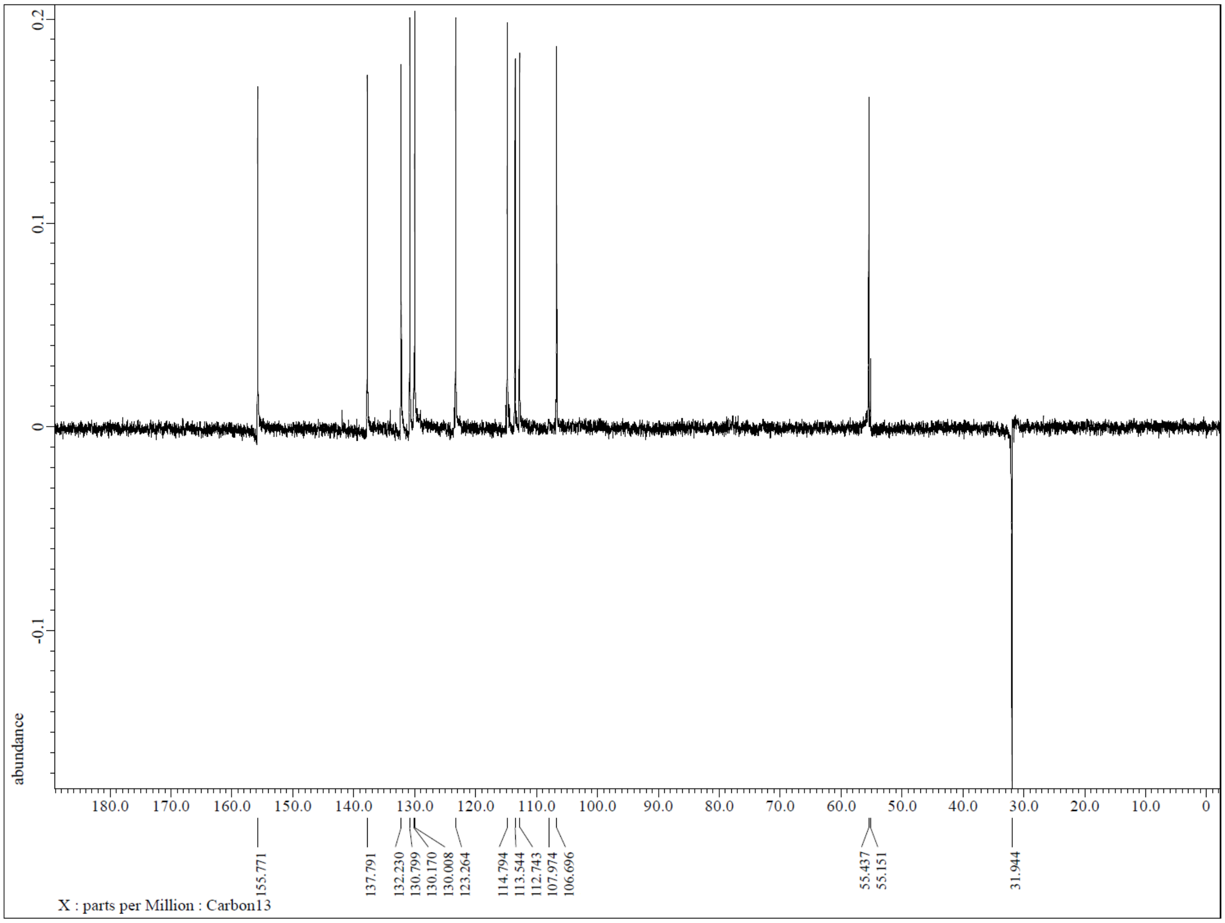

Figure S7 DEPT-135 NMR spectrum of **5g**

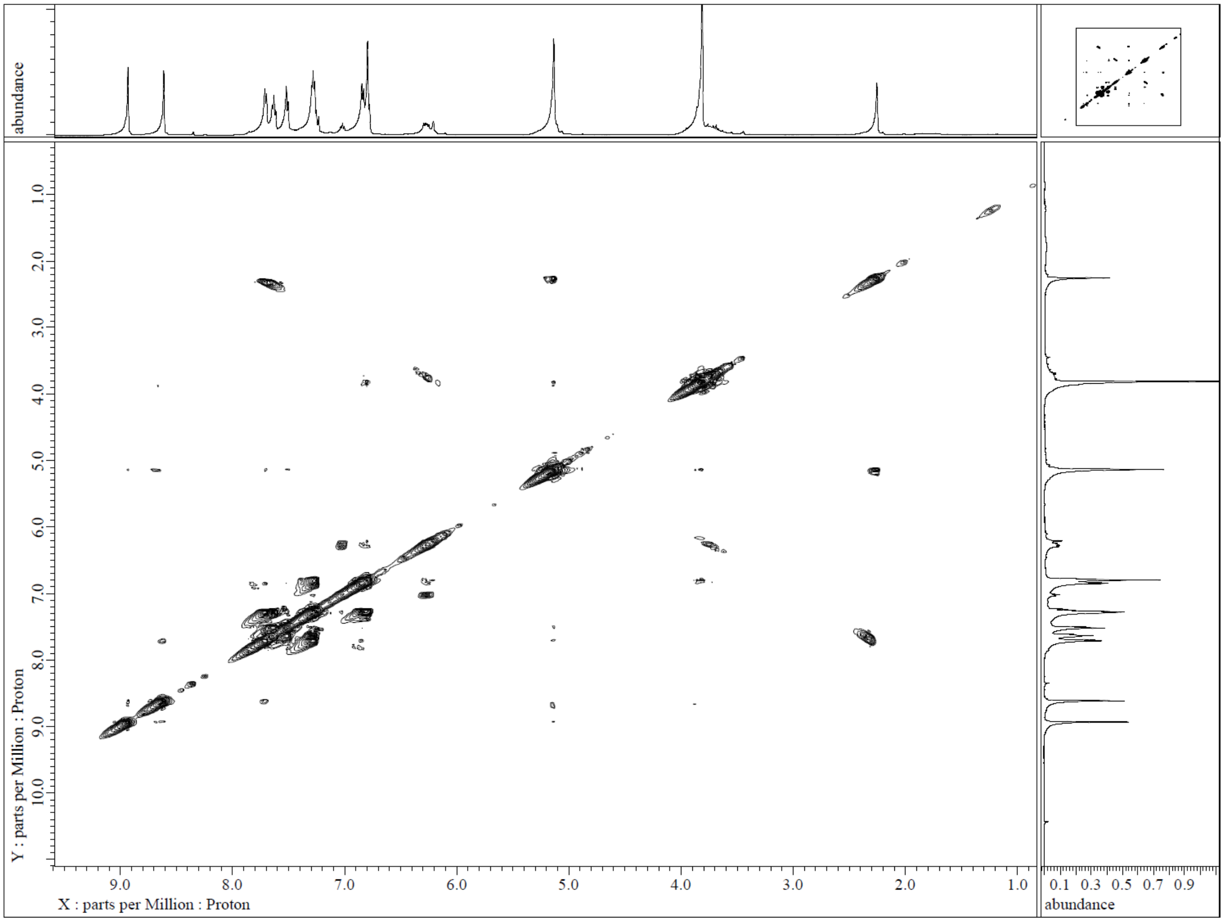

Figure S8 ^{1}H,^{1}H-COSY NMR spectrum of **5g**

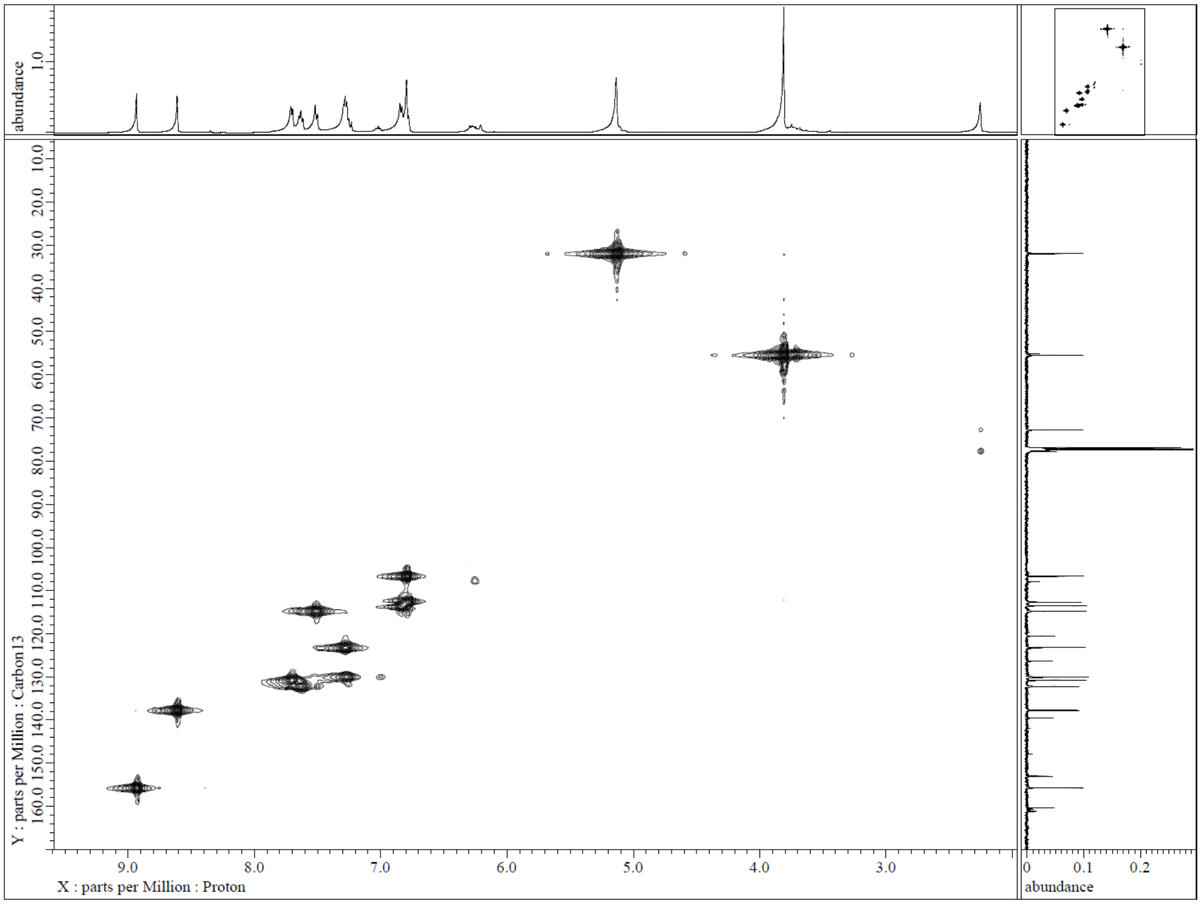

Figure S9 ¹³C,¹H-COSY NMR spectrum of **5g**

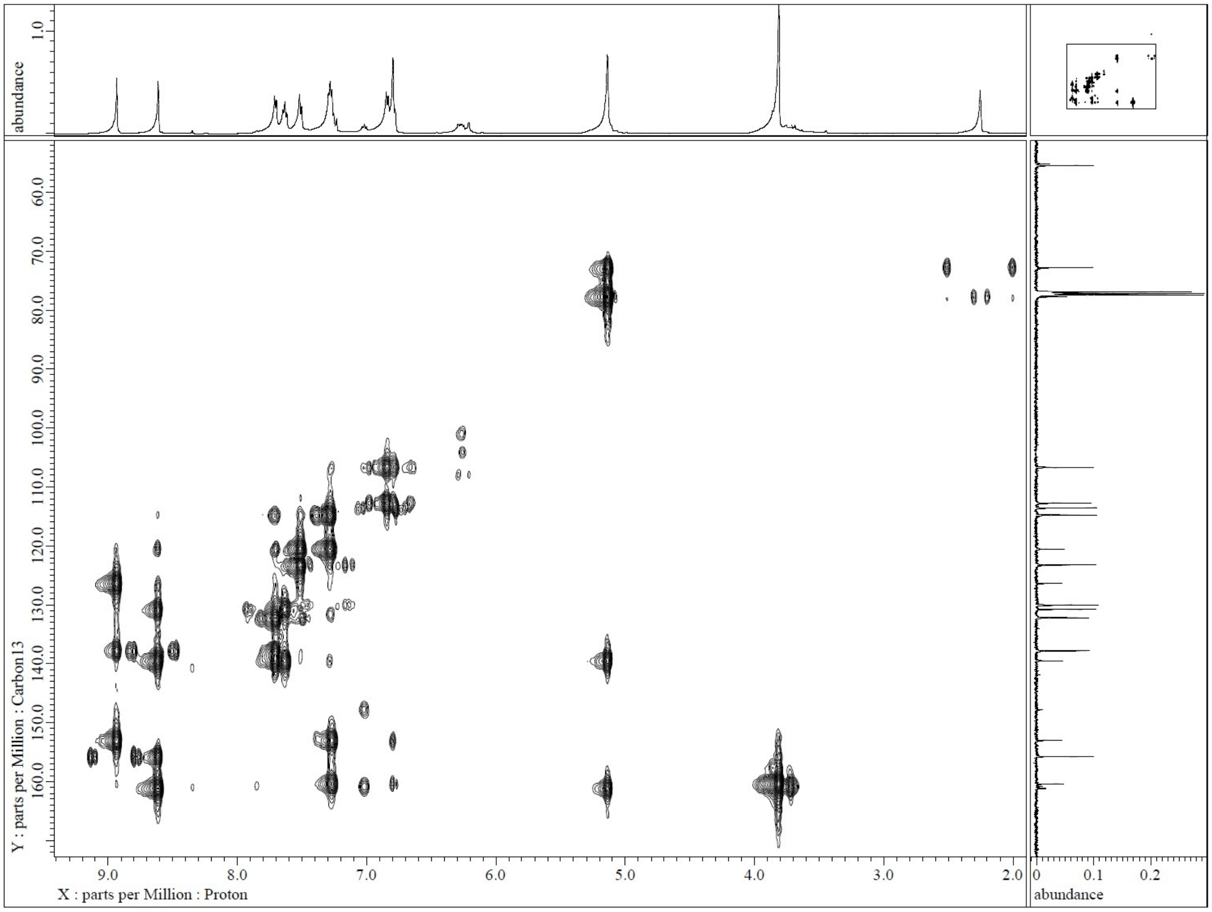

Figure S10 HMBC spectrum of **5g**

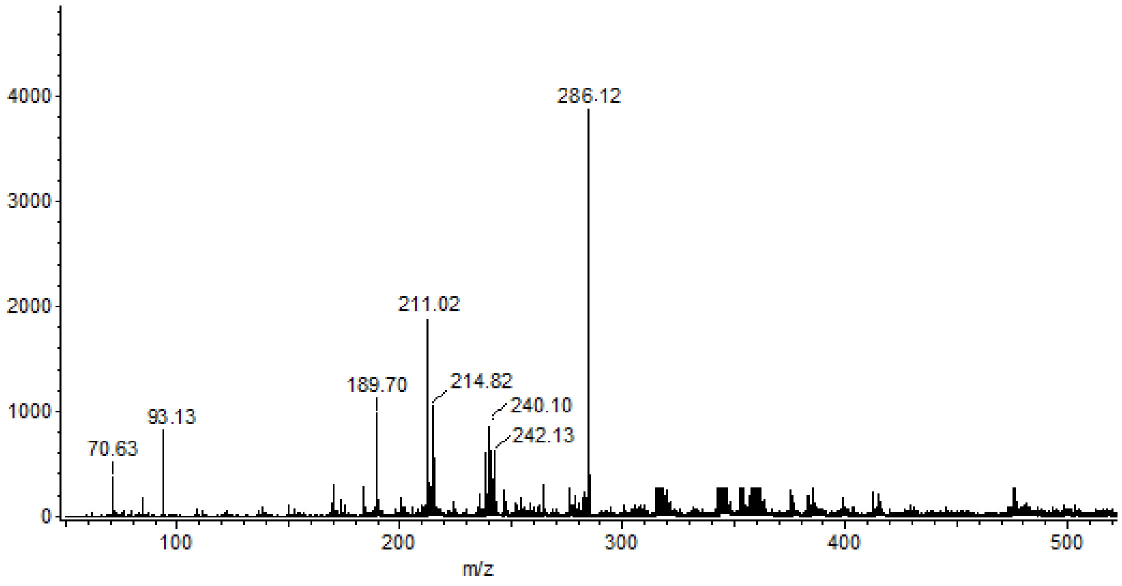

Figure S11. Mass spectrum of **5a**

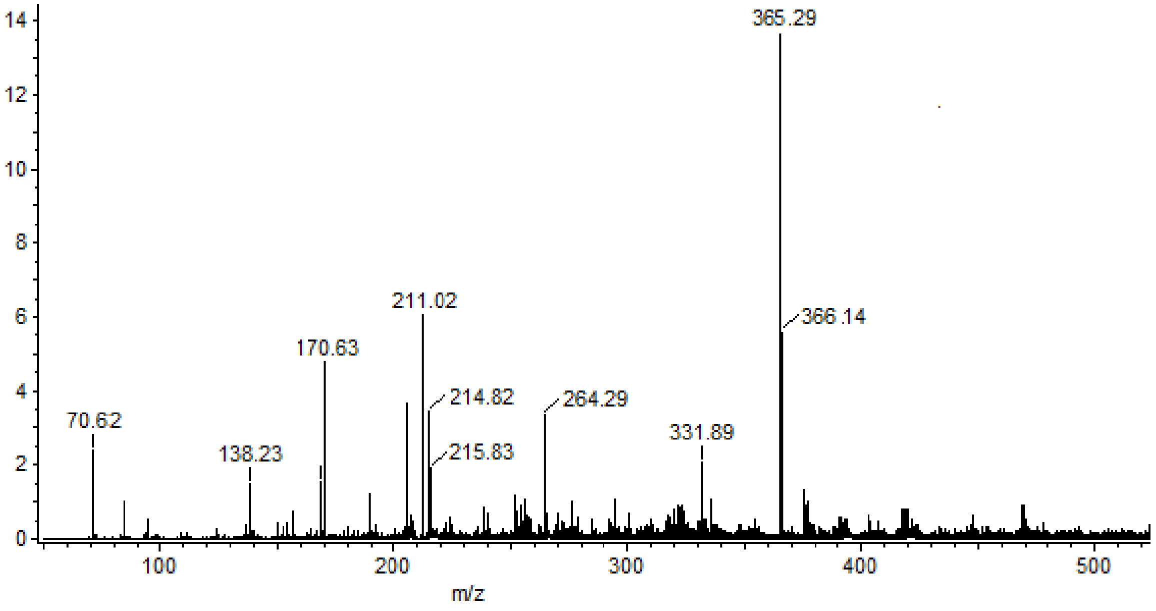

Figure S12. Mass spectrum of **5b**

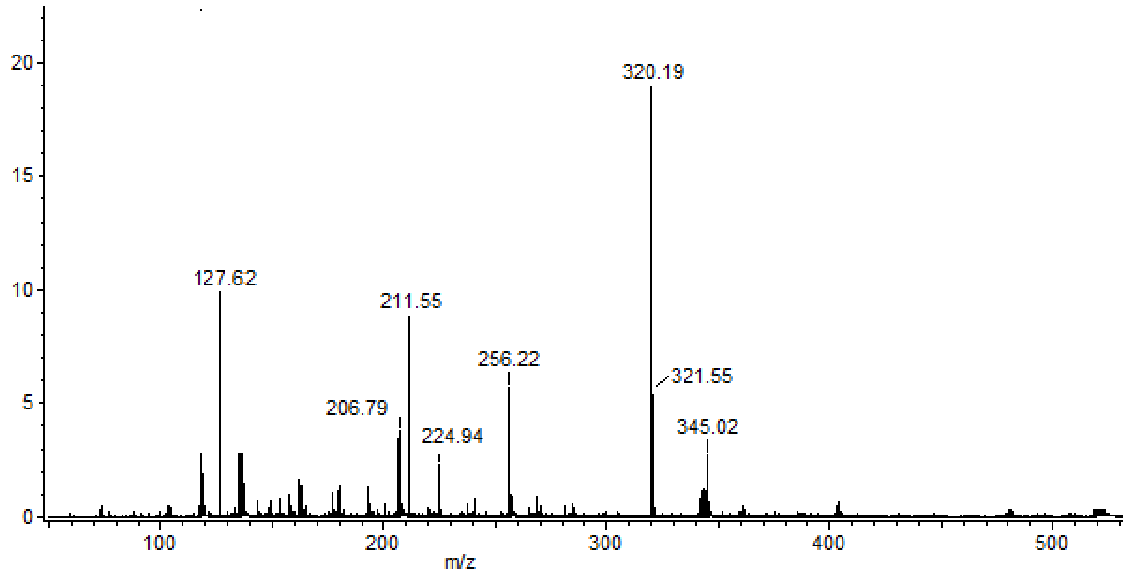

Figure S13. Mass spectrum of **5c**

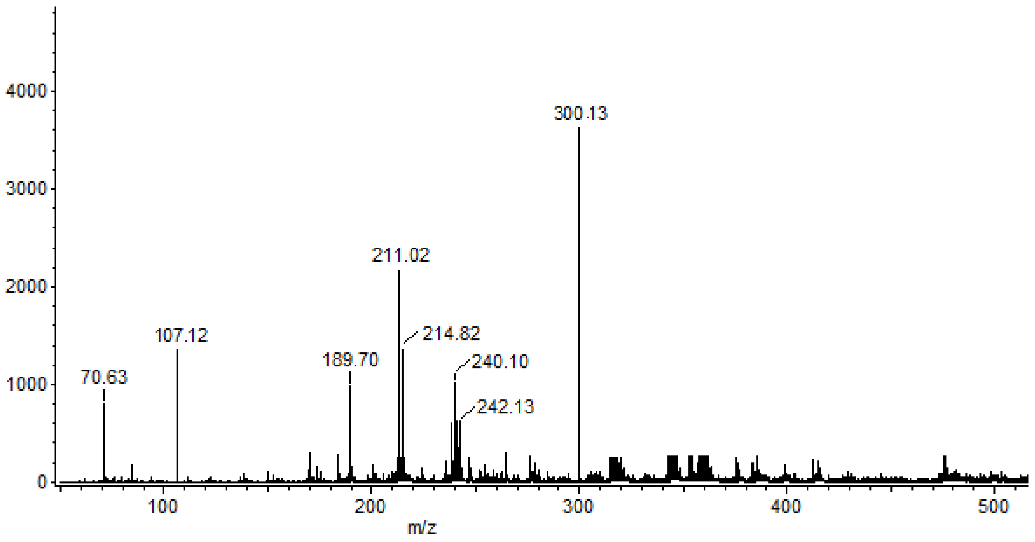

Figure S14. Mass spectrum of **5d**

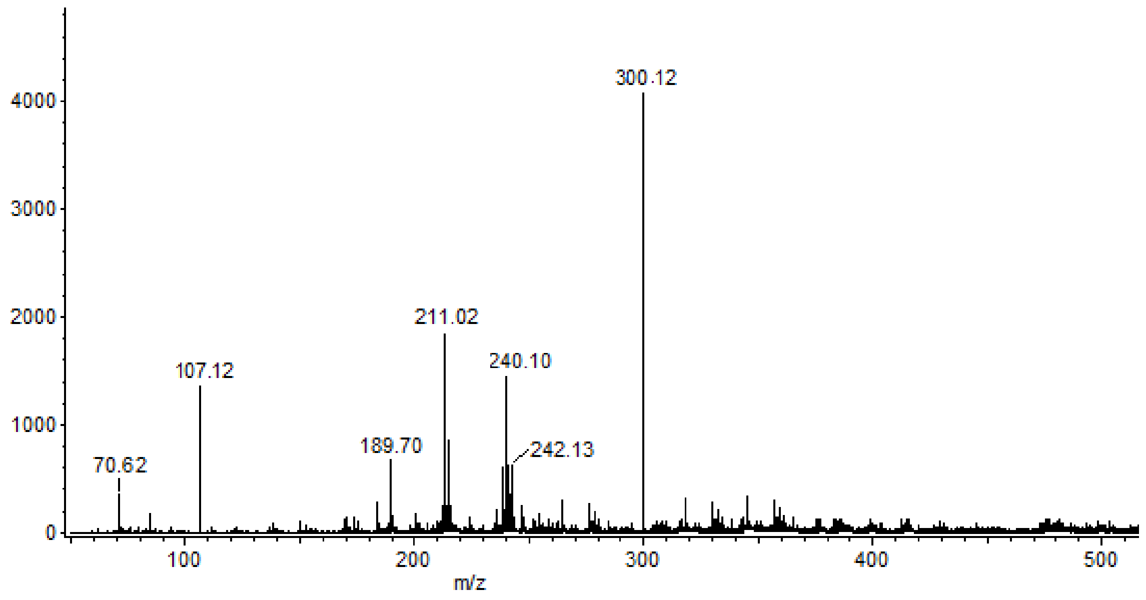

Figure S15. Mass spectrum of **5e**

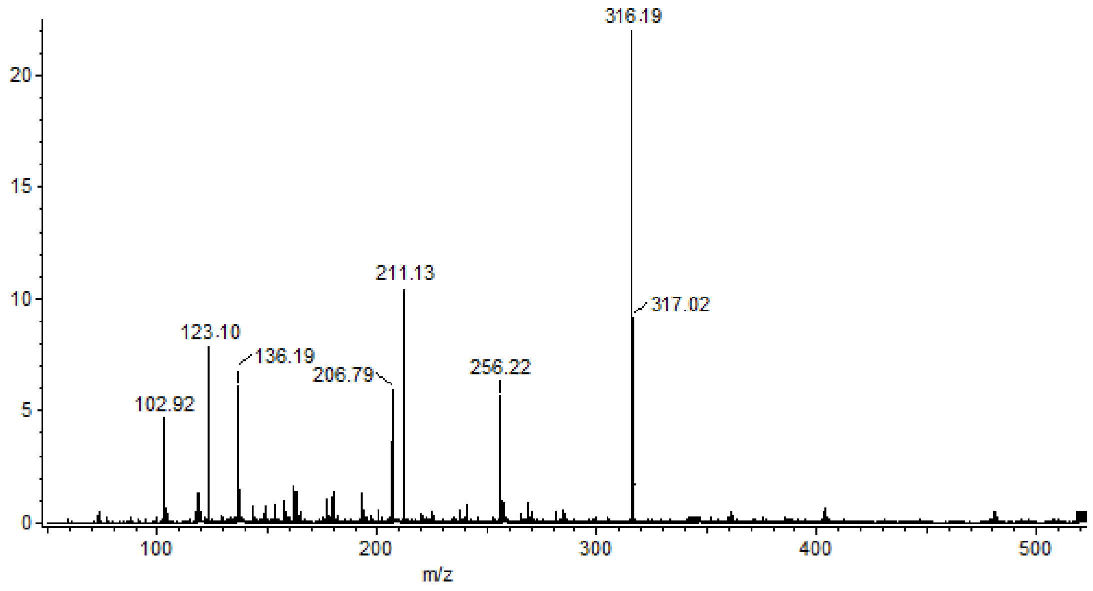

Figure S16. Mass spectrum of **5f**

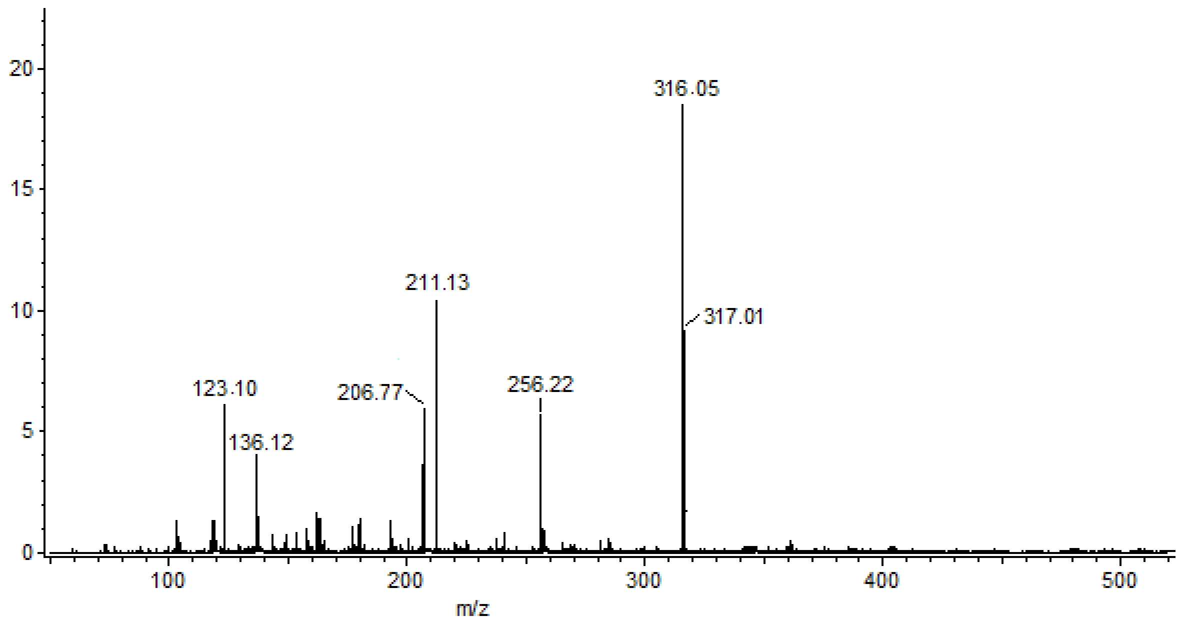

Figure S17. Mass spectrum of **5g**

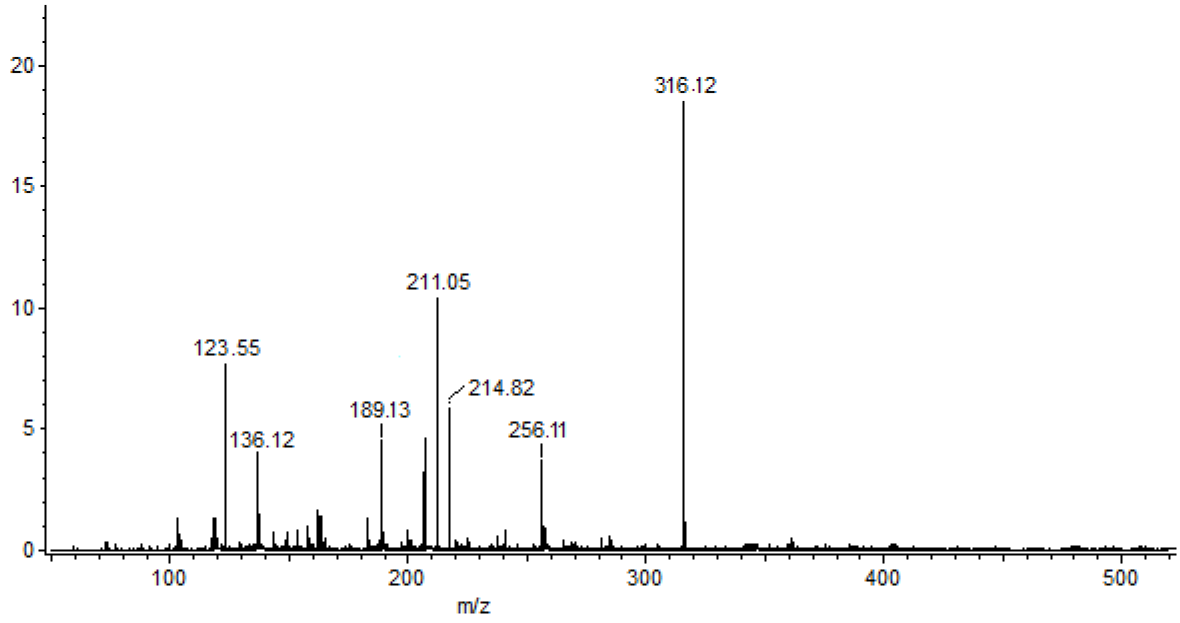

Figure S18. Mass spectrum of **5h**
